# Engineering Nitric Oxide-Releasing Antimicrobial Dental Coating for Targeted Gingival Therapy

**DOI:** 10.1021/acsabm.4c00051

**Published:** 2024-04-09

**Authors:** Manjyot Kaur Chug, Natalie Crutchfield, Mark Garren, Hitesh Handa, Elizabeth J. Brisbois

**Affiliations:** School of Chemical, Materials, and Biomedical Engineering, University of Georgia, 302 E Campus Rd, Athens, Georgia 30605, United States

**Keywords:** nitric oxide, dental pathogens, biofilm, periodontitis, antimicrobial coating

## Abstract

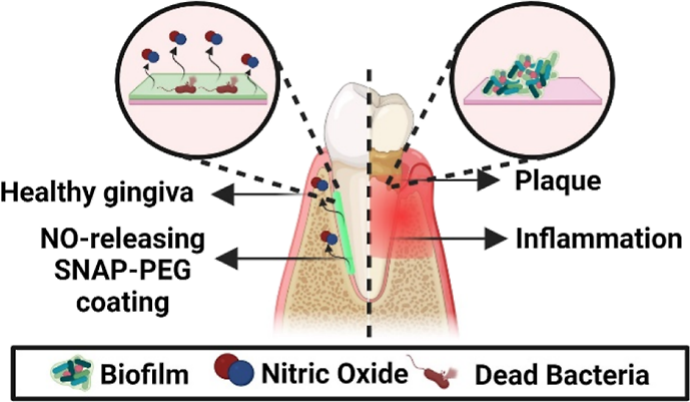

Bacterial biofilms play a central role in the development and progression of periodontitis, a chronic inflammatory condition that affects the oral cavity. One solution to current treatment constraints is using nitric oxide (NO)—with inherent antimicrobial properties. In this study, an antimicrobial coating is developed from the NO donor *S*-nitroso-*N*-acetylpenicillamine (SNAP) embedded within polyethylene glycol (PEG) to prevent periodontitis. The SNAP-PEG coating design enabled a controlled NO release, achieving tunable NO levels for more than 24 h. Testing the SNAP-PEG composite on dental floss showed its effectiveness as a uniform and bioactive coating. The coating exhibited antibacterial properties against *Streptococcus mutans* and *Escherichia coli*, with inhibition zones measuring up to 7.50 ± 0.28 and 14.80 ± 0.46 mm^2^, respectively. Furthermore, SNAP-PEG coating materials were found to be stable when stored at room temperature, with 93.65% of SNAP remaining after 28 d. The coatings were biocompatible against HGF and hFOB 1.19 cells through a 24 h controlled release study. This study presents a facile method to utilize controlled NO release with dental antimicrobial coatings comprising SNAP-PEG. This coating can be easily applied to various substrates, providing a user-friendly approach for targeted self-care in managing gingival infections associated with periodontitis.

## 1 Introduction

Periodontitis affects a significant portion of the global population, with more than 40% of adults in the United States affected by the condition, despite many of these oral health issues being preventable. This chronic inflammatory disease has a prevalence of approximately 11.2% globally, ranking it as the sixth most common human disease despite this oral health issue being preventable.^1,2^ Periodontal disease is characterized by aggregation of bacteria in the periodontal pocket within the gingival that begins as gingivitis and progresses to periodontitis. This inflammatory condition leads to the degradation of the tissues supporting the teeth, ultimately resulting in tooth loss and affecting speech, nutrition, and aesthetics. Moreover, periodontitis has systemic implications, contributing to conditions such as cardiovascular disease and coronary heart disease by promoting systemic inflammation. Once advanced to periodontitis, there is significant soft tissue damage and alveolar bone loss.^3^ Severe periodontitis is one of the world’s most prevalent inflammatory diseases. There is a predictable increase in the healthcare burden of periodontal disease as the population and life expectancy are increasing.^4^ Considering the indirect costs, in 2018, periodontal disease caused an estimated loss of $154.06B in the US and €158.64B in Europe.^5^ The current point of care for periodontitis includes scaling, root planning, and antibiotics in the form of mouthwashes and gels, which have limited reach to the periodontal pockets, and microperiodontal surgery.^2^ Treatment for periodontitis requires clinical intervention, leading to significant economic costs and healthcare disparities. Therefore, creating an accessible prevention method for periodontal disease, such as bioactive coatings for interdental cleaning (i.e., dental floss), is of interest for at-home care. Such technologies have the potential to improve patient accessibility and compliance, thus improving general health outcomes.

Interdental cleaning using dental floss is shown to mechanically remove plaque in interproximal regions of the oral cavity, thus actively flossing lowers the risk for dental caries and periodontal disease.^6^ However, improper brushing or flossing techniques reduce the mechanical capacity to remove plaque.^7^ Furthermore, patients with hindered motor function or any other disability that often struggle with dental hygiene would otherwise benefit from the addition of antiseptic agents combined with the mechanical action of flossing.

Coatings have proven effective on materials like floss and sutures, showing antimicrobial activity against bacteria such as *Porphyromonas gingivalis* and *Enterococcus faecalis*.^8^ Additionally, antibiotics incorporated into floss for targeting bacteria in periodontal pockets are well established. Controlled release of antimicrobial agents at the infection site is crucial for optimal efficacy and biocompatibility. Kaewiad et al. impregnated floss with povidone-iodine coated with Eudragit L-100 as an antimicrobial agent against periodontitis-associated bacteria, including *P. gingivalis*, *Aggregatibacter actinomycetemcomitans*, and *Prevotella intermedia*.^9^ However, research indicates potential toxicity linked with povidone-iodine coating, capable of causing irritation or sensitivity.^10^ Similarly, various antibiotics and antiseptics in dentistry, like chlorhexidine, cetylpyridinium chloride/bromide, amoxicillin, clindamycin, and azithromycin, face challenges due to the complex biofilm matrix of periodontal disease, reducing their efficacy and susceptibility to resistance.^11,12^ Moreover, chemical agents such as cetylpyridinium chloride/bromide in mouthwashes may not effectively target all bacterial types, leading to incomplete control. The implementation of more accessible treatments is vital, as many dental diseases can be prevented but may remain untreated due to limited patient access or affordability. Dental biofilms or plaque are closely associated with dental caries and periodontal disease, with periodontitis involving multifactorial infections by a broad spectrum of bacterial species releasing inflammatory mediators.^13^ Hence, alternative strategies for addressing antibiotic resistance and toxicity are crucial for effective and sustainable therapy.

One solution to these constraints of current treatment is the use of nitric oxide (NO), an endogenously produced gaseous molecule that has antibacterial properties through several mechanisms, including nitrosation of amines and thiols, chemical manipulation of DNA, lipid peroxidation, promotion of iron depletion, and tyrosine nitrosation.^14−16^ The capacity of NO has been demonstrated to eliminate a broad range of bacteria, including both Gram-negative and -positive bacteria.^17^ The short half-life of NO is in the order of seconds; thus, these mechanisms occur quite rapidly, preventing bacteria from becoming resistant to NO.^18^ NO is naturally produced by endothelial cells for many metabolic pathways, including immune response.^19^ However, NO’s instability and short biological half-life in physiological conditions require a donor molecule to stabilize the NO release. There is a pharmacologically active class of NO donors, such as nitrates, *N*-diazeniumdiolates (NONOates), and *S*-nitrosothiols (RSNOs), which can be integrated with a variety of medical-grade polymers for prolonged and controlled NO release.^20−22^*S*-nitroso-*N*-acetylpenicillamine (SNAP) is an RSNO donor catalyzed by light, heat, and metal ions widely used to sustain a controlled NO release.^23,24^ The ease of synthesis, stability under physiological conditions, biocompatibility, and tunable release kinetics make SNAP a more appropriate RSNO donor for this application compared to GSNO (short half-life) and NONOates (cytotoxicity concerns). The *S*-nitrosothiol bond is cleaved and forms a nontoxic disulfide adduct and NO.^25^ Studies have shown that SNAP can be further stabilized by incorporation into polymer matrixes via internal hydrogen bonding, which can prolong the NO release.^26^ Previous studies have shown the addition of SNAP to polyethylene glycol (PEG) increases the duration of NO release and stabilizes the release kinetics compared to SNAP in the dry crystalline solid state.^27^ Similarly, other RSNO donors have been incorporated into PEG matrices for targeted topical delivery and shown to reduce the rates of photochemical and thermal release of NO compared to aqueous solutions.^28,29^

The emergence of NO-releasing materials as a superior alternative to synthetic antibacterial compounds is attributed to the lack of known bacterial resistance to NO, coupled with their broad-spectrum antibacterial activity and biocompatibility.^30^ Therefore, inspired by the promising antimicrobial properties of NO, in this study, a PEG hydrogel coating blended with SNAP (SNAP-PEG) for dental floss is used for local delivery of NO into the periodontal pocket to prevent subgingival infection associated with periodontitis.^31−33^ The concept is based on the idea that the incorporation of PEG extends the bioavailability of NO by shielding SNAP from premature degradation. This enables SNAP-PEG to effectively combat potential pathogens. Presented here is a straightforward method for developing a dental floss coating that facilitates controlled local NO release, aiming to prevent periodontitis by eliminating bacterial pathogens. Controlled NO-releasing floss not only improves the biological response to antimicrobial agents but also provides therapeutic effects. The fabrication of the floss coating, surface characterization, efficiency of indirect drug delivery, kinetics of NO release, degradation of the SNAP-PEG coating, and shelf-life studies were conducted. Furthermore, in vitro assessments were performed to evaluate the antimicrobial activity and biocompatibility. The antimicrobial activity was measured against *Streptococcus mutans* and *Escherichia coli* to illustrate the broad-spectrum nature of the coating. The floss was exposed to human gingival fibroblast and osteoblast cells for a biocompatibility assessment. This innovative solution of targeted delivery of antibacterial agents through NO-releasing floss holds promise for improving the effectiveness of treatment and overcoming challenges associated with the current traditional approaches in addressing gingival infections.

## 2 Materials and Methods

### 2.1 Materials

Ethylenediamine-tetraacetic acid (EDTA), methanol (MeOH), potassium phosphate dibasic, sodium nitrite, sulfuric acid (H_2_SO_4_), 3-(4,5-dimethylthiazol-2-yl)-2,5-diphenyltetrazolium bromide (MTT), Luria–Bertani (LB) broth and agar, and both 3350 and 1000 average molecular weight polyethylene glycol were purchased from Sigma-Aldrich (St. Louis, MO, USA). Hydrochloric acid (HCl) and fetal bovine serum (FBS) were purchased from VWR (Radnor, PA, USA). All buffers and other aqueous solutions were prepared using (18.2 MΩ) Ultrapure water using an in-house distillation system from Mettler Toledo (Columbus, OH, USA). Phosphate-buffered saline (0.01 M PBS) containing 2.7 mM KCl, 138 mM NaCl, 1.8 mM KH_2_PO_4_, and 10 mM Na_2_HPO_4_ at pH 7.4 was used in all experiments, unless otherwise stated. Brain heart infusion agar and broth were purchased from McKesson Medical-Surgical (Irving, TX, USA). *S. mutans* (ATCC 25175) and *E. coli* (ATCC 25922) were purchased from the American Type Culture Collection (ATCC, Manassas, VA, USA). Human-derived osteoblast cell line hFOB 1.19 (ATCC CRL-11372), primary gingival fibroblasts (HGF) (ATCC PCS-201-018), fibroblast basal medium, and the associated fibroblast growth kit with low serum were also purchased from ATCC. Dulbecco’s modified Eagle’s medium with nutrient mixture F12 (1:1 by volume) was purchased from Thermo Fisher Scientific (Waltham, MA, USA). Trypsin–EDTA and sterile phosphate-buffered saline (0.01 M) without calcium and magnesium was obtained from Corning (Corning, NY, USA). REACH unflavored waxed nylon floss and Angzhili Dental removable teeth model with silica gel gingival were purchased from Amazon.

### 2.2 *S*-Nitroso-*N*-acetylpenicillamine Synthesis

*S*-Nitroso-*N*-acetylpenicillamine (SNAP) was synthesized in a slightly modified procedure based on prior work.^34,35^ The synthesis begins with the nitrosation of *N*-acetylpenicillamine (NAP) with an excess molar ratio of sodium nitrite in deionized water. The NAP was added to the MeOH and concentrated HCl and H_2_SO_4_ which was stirred until the NAP was fully dissolved (1–2 min). Then, NaNO_2_ was added dropwise (<10 min) and finally the mixture was chilled while N_2_ gas was blown over the solution to allow the SNAP crystals to precipitate out over 8 h. The green crystalline product is finally filtered and dried while being protected from light exposure. The purity of SNAP was determined with a nitric oxide analyzer by injecting 30 μL 50 mM CuCl_2_ and 1.5 μL 10 mM cysteine to PBS (without EDTA) for a total of 5000 μL then injecting 25 μL of SNAP and allowing the NO release to exhaust. This was repeated at least 3 times, and the release profiles were then integrated to determine the total NO release compared to the theoretical yield. Purity was also confirmed with the ^1^H NMR spectra of NAP and SNAP used in the coatings (Figure S1). All SNAP used was ≥90% pure.

### 2.3 Development of SNAP-PEG Coating

The NO-releasing coatings were constructed to incorporate the antimicrobial properties of NO into dental floss for the prevention of gingival infections. For this formulation, PEGs with average molecular weights of 1000 and 3350 were combined, based on previous reports, in a 1:2 ratio in MeOH (2500 mg mL^–1^).^36^ The combination of the different molecular weights of PEG causes the coating to be waxy and can easily be deposited by mechanical agitation. The solution was then heated to 60 °C until the mixture was homogeneous, and once cooled, different weight percents of SNAP (1, 5, and 10 wt %) were added to the PEG/MeOH solution. A commercially available nylon floss was cut into approximately 8 cm strips and secured at one end. The samples were then dip coated by the different SNAP wt % solutions three times with intervals of one min to allow for the coating to cool and adhere to the nylon substrate. The samples were allowed to thoroughly dry for 24 h at room temperature undisturbed to facilitate the complete evaporation of MeOH. This precautionary measure aimed to prevent any interference from MeOH in subsequent studies, ensuring that only the influence of the SNAP-PEG coating was observed. For control samples (CTRL), the PEG formulation was dissolved in methanol without the addition of SNAP and used to coat the floss samples following the same fabrication and drying methods.

### 2.4 Determination of Amount of SNAP on Floss Using UV–Vis Spectroscopy

The individual coated samples were cut into 1 cm sections and then suspended and agitated in 1 mL of PBS buffer containing 100 μM EDTA to dissolve the SNAP-PEG coating. The concentration of SNAP was determined using a UV–vis spectrophotometer (Cary 60, Agilent Technologies). The molar absorptivity of SNAP in PBS containing EDTA at 340 nm was determined to be 840 M^–1^ cm^–1^. The PEG mixture without SNAP was used as a blank control to confirm that the absorbance peak spectra at 340 nm are due to the presence of SNAP. The absorbance is then used to determine the weight of SNAP present in the samples from standard curve data of the SNAP used in synthesis.

### 2.5 Total SNAP-PEG Coating Weight Uniformity

To quantify the uniformity of the coating, weight distribution samples were cut into 1 cm sections and weighed. Then, the coating was fully dissolved in 1 mL of PBS with agitation and then dried, and the floss was weighed again. The weight of the floss sample and coating combined was subtracted from the weight of the cleaned floss to find the weight of the coating. Data from the study are presented as the weight of the SNAP-PEG coating was measured in mg, and the mean and standard deviation for the segments (control, 1, 5, and 10 wt % SNAP-PEG coated samples) with 16 replicates were obtained.

### 2.6 Indirect Drug Delivery Efficiency

To determine drug delivery efficiency into the periodontal pocket, via a slightly modified method,^12^ the tooth model was lightly lubricated with PBS 10 mM containing 100 μM EDTA, and then individually coated floss samples (1 cm segments with 6 mg total coating) were passed back-and-forth three times in the periodontal pocket in either two of the front incisors of a tooth model. The tooth model was then swabbed in the periodontal pocket using a cotton-tipped stick that had been slightly wet with PBS containing 100 μM EDTA and placed back in a tube of PBS containing EDTA (1 mL) and agitated with a vortex device to dissolve and homogenize the floss coating. The SNAP concentration was then calculated by measuring the absorbance at 340 nm using a UV–vis spectrophotometer. The molar absorptivity at 340 nm was determined to be 1002 M^–1^ cm^–1^. The weight is reported as average milligrams of SNAP deposited in the periodontal pocket (*N* = 4).

### 2.7 Surface Characterization

Images were collected using scanning electron microscopy (SEM) from Thermo Fisher, the Teneo FE-SEM with a current of 0.4 nA and the accelerating voltage was 5.0 kV. Samples were coated with 20 nm of gold–palladium with a Leica sputter coater before SEM imaging. The SEM images illustrate the effectiveness of the deposition of the coating after flossing. Images illustrate the coating before and after the sample has been deposited in the periodontal pocket of a tooth model. A medical tooth model with malleable silicone rubber gingival and high-density polyethylene teeth was used to mimic the deposition of the coating between the teeth into the periodontal pocket in representational 3-dimensional space. Additionally, an energy-dispersive X-ray spectroscopy system (EDS, Oxford Instruments) with an accelerating voltage of 10 kV was run in conjunction to perform the elemental analysis of the surface of the floss coating. Sulfur measurements corresponded to the presence of SNAP or the parent thiol.

### 2.8 NO Release Studies

#### 2.8.1 Total Nitrite Concentration to Estimate NO Concentration

To illustrate the decomposition of the SNAP-PEG coating in solution the NO concentrations were estimated via Griess assay by finding the total nitrite concentration present.^37,38^ To estimate NO concentrations via the Griess assay, 0.5 cm floss samples were submerged in 0.1 mM PBS containing 100 μM EDTA (1 mL) and incubated without agitation at 37 °C for 2, 4, 6, and 30 h. Aliquots (10 μL) of this sample were added to the Griess reagent (90 μL, 22.22 mg mL^–1^) for a final concentration of 20 mg mL^–1^ to form a colorimetric product, and the absorbance measured in each well was read at 540 nm with a plate reader (BioTek Cytation 5 imaging reader). Sodium nitrite standards (0, 0.3125, 0.625, 1.25, 2.5, 5, 10, 20, 40 mM) were used to normalize the assay reactivity and associated absorbance. The molar absorptivity at 540 nm was determined to be 1240 M^–1^ cm^–1^.

#### 2.8.2 Nitric Oxide Analyzer

The experimentation was carried out to evaluate the NO release profile of each wt % SNAP-PEG-coated floss. Instantaneously, the NO-release from the floss samples was measured by a Sievers 280i chemiluminescence Zysense Nitric Oxide Analyzer (NOA) 280i instrument (Frederick, CO, USA). The NOA was calibrated using a two-point calibration (0 and 45 ppm of NO calibration gas). The NOA had a cell pressure of 6.4 Torr and a supply pressure of 10.9 Torr. The initial baseline NO release was in the range of 0–1 ppb. Given that the oral cavity maintains a pH close to neutrality (6.7–7.5) through saliva, the release of NO from samples was assessed mimicking these conditions.^39^ Samples were prepared by adding 200 μL of PBS buffer with 100 μM EDTA (10 mM, 7.4 pH) solution to a KimTech wipe to keep the sample moist. These conditions emulate the physiological environment of the human mouth, with the samples incubated at 37 °C and suspended to avoid direct contact with the KimTech wipe. The NO released from the coated floss samples was immediately swept to the chemiluminescence detection chamber due to the flow of nitrogen gas (200 mL min^–1^). The samples were incubated at 37 °C between time points, and the NO release was measured at 0, 2, 6, and 30 h to find the average ppb of NO for each sample. The NO flux is then determined by subtracting the baseline from the NOA from the release profile of the sample and converting the ppb to flux (×10^–10^ mol min^–1^ cm^–2^), normalizing the samples by surface area.

### 2.9 Shelf Life Stability of SNAP-PEG Coating

To evaluate the stability of the SNAP-PEG coating, the coated floss samples were wrapped in a KimTech wipe and stored at room temperature (24 °C) in an airtight vial with desiccant protected from ambient light. The amount of remaining SNAP in the coating was measured using the same UV–vis spectroscopy method as described in Section 2.4 to determine the absorbances of the samples compared to the initial levels of SNAP present. The samples were evaluated at four different time points (7, 14, 21, and 28 d) to determine the stability of the samples over a 28 d period in relevant medical storage conditions. The data are reported as the percent SNAP remaining on the coated floss after each time point normalized to the initial percent of SNAP in freshly prepared samples on day 0.

### 2.10 Antimicrobial Activity of SNAP-PEG Coating

The effectiveness of the coating in eradicating bacteria was assessed against two distinct strains: *S. mutans* (a Gram-positive cocci associated with periodontal infections) and *E. coli* (a Gram-negative rod commonly linked to infections related to medical devices). Although *S. mutans* has traditionally been identified as a primary causative agent in periodontal infections, recent research indicates a connection between *E. coli* and osteomyelitis in diabetic patients with aggressive bilateral maxillary necrosis.^40^ Both of these bacteria are classified as opportunistic pathogens and are often associated with infections characterized by the formation of biofilms, which can lead to severe systemic infections.^41^ The antibacterial efficacy of the NO-releasing SNAP-PEG coating was evaluated using a zone of inhibition study. For this, *E. coli* was grown in LB media and *S. mutans* in BHI media until the mid-log phase and then diluted in sterile 10 mM PBS. The bacteria suspension was washed by centrifuging the culture at 4400 rpm for 7.5 min and resuspended into sterile PBS. Bacteria OD_600_ was measured using a UV–vis spectrophotometer (Cary 60, Agilent Technologies). The bacteria culture was diluted to 0.1 OD and 100 μL of suspension was pipetted onto an agar plate and uniformly spread using a sterile cotton swab. Segments of floss with 6 mg of the coating were evenly positioned on the plate with the SNAP-PEG coating at various weight percentages (0,1, 5, and 10 wt %). The plates were incubated overnight at 37 °C and then the zone of inhibition was measured by finding the area of the zone of impeded bacterial growth surrounding the floss with ImageJ analysis. The results are presented as the average mm^2^ of zone ± standard deviation (*n* ≥ 6) with three independent biological replicates.

### 2.11 Cytocompatibility Assessment

#### 2.11.1 Cell Culture Preparation

Cells were revived from cryopreserved stocks using complete media for each cell line following the manufacturers’ recommendations. HGF cells were cultured in fibroblast basal medium supplemented with a growth kit containing fetal bovine serum (2%), ascorbic acid (50 μg/mL), recombinant human insulin (5 μg/mL), hydrocortisone hemisuccinate (1 μg/mL), recombinant human fibroblast growth factor b (5 ng/mL), l-glutamine (7.5 mM), and penicillin–streptomycin (10 U/mL). The hFOB cells were grown in media containing an equal volume of Ham’s F12 medium to Dulbecco’s modified Eagle’s medium, further supplemented with l-glutamine (2.5 mM), fetal bovine serum (10%), and G418 antibiotic (0.3 mg/mL). Cells were incubated at 37 °C under a 5% CO_2_-humidified atmosphere. Cells were treated with clean media every 48 h and grown to no greater than 80% subconfluency. For the controlled release leachate testing, cells were detached via enzymatic treatment with trypsin (0.05% supplemented with 5 mM EDTA), centrifuged to collect the cell pellet (200 RCF, 5 min), and resuspended to achieve a seeding density of 10,000 cells/well in culture-treated 96-well plates.

#### 2.11.2 Controlled Release Study for Cytocompatibility

Cytotoxicity screening of SNAP-PEG coated substrates was carried out following the International Organization for Standardization Protocol 10993-5:2009 with minor deviation.^42^ In brief, floss segments with 6 mg of the different formulations of the PEG coating were dissolved in 1 mL of complete media for 24 h. Concurrently, cells are seeded onto 96-well plates (100 μL of complete media per well) and incubated for 24 h. Afterward, leachate samples from each substrate classification are used to treat cells, replacing media with leachate-containing media (100 μL). Control wells were concurrently developed using clean complete media. Cells were grown for an additional 24 h. Following incubation, media was aspirated off from each well and replaced with MTT-containing media (0.5 mg/mL MTT, 100 μL). Cells were incubated for an additional 2 h. Subsequently, wells were aspirated of undissolved tetrazolium salt, and the remaining formazan salt was dissolved in dimethyl sulfoxide (200 μL/well). Wells were read for the absorbance at 570 nm with a reference reading at 690 nm. The percent cellular viability was then calculated relative to the control wells as follows
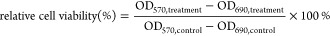
1

Final data are reported as the mean cellular viability ±standard deviation (SD) (*N* = 4 technical repeats across three independent passages).

### 2.12 Statistical Analysis

The statistical analysis for all the reported studies was done in GraphPad Prism 9 (GraphPad Software, San Diego, CA, USA). For the NO flux determination, a 2-way ANOVA with Tukey’s multiple comparison test with an alpha value of 0.05 was used to determine any significant differences between the compositions. Additionally, for the remaining studies, comparisons of the different weight percentages of SNAP were determined by an ordinary one-way analysis of variance (ANOVA) with Tukey’s method for multiple comparisons, given values of *p* < 0.05 to be significantly different.

## 3 Results and Discussion

### 3.1 Development of NO-Releasing Floss Coating

**Figure 1:**
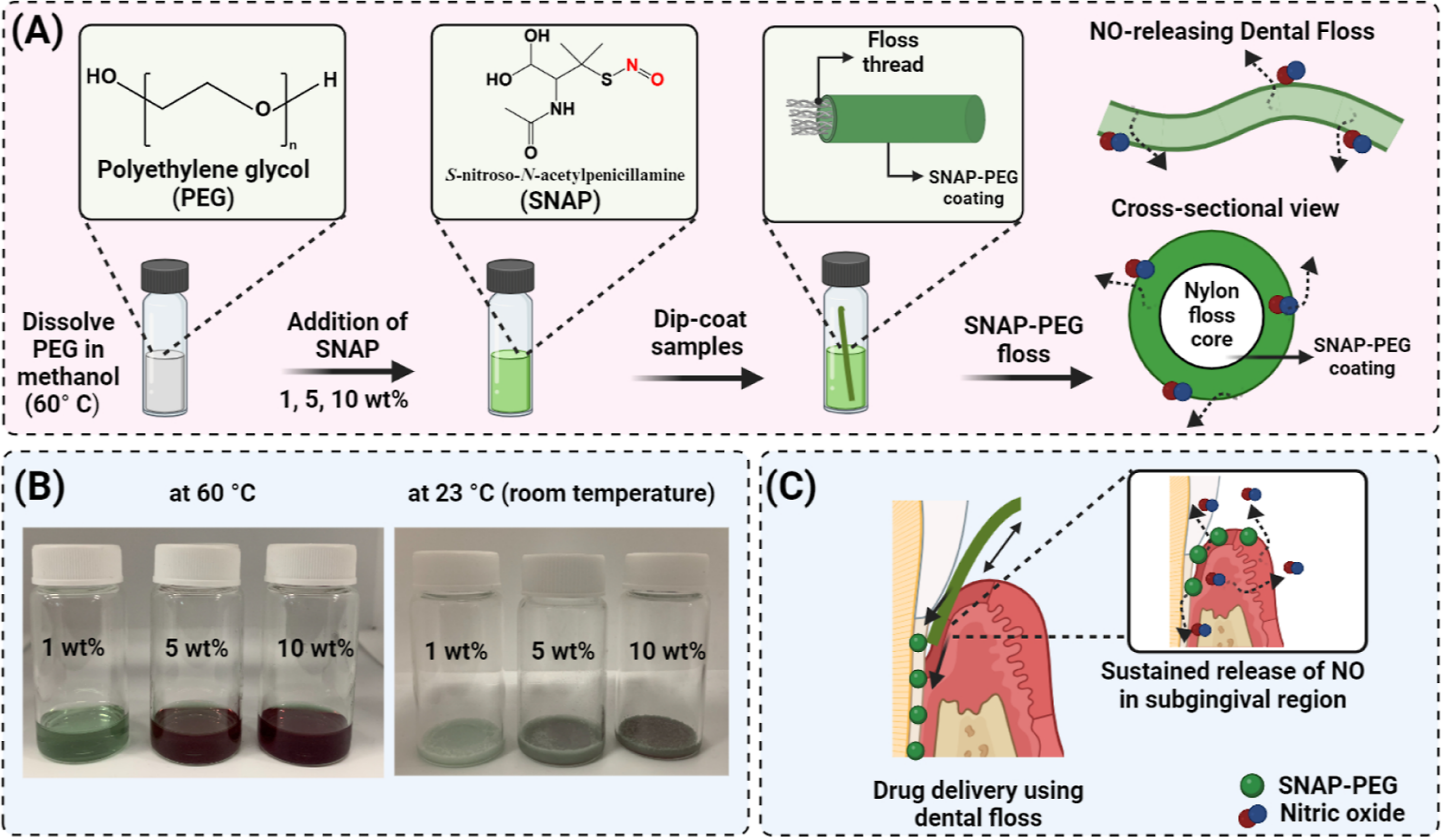
Schematic representation of NO-releasing dental floss for advanced care of the subgingival region. (A) NO-releasing floss is fabricated using a SNAP-PEG mixture coating. Various amounts of SNAP can be loaded into the mixture to enhance the tunability of NO release on the surface. (B) The versatility of the SNAP-PEG mixture is shown as solutions when heated to 60 °C and as a solid substrate at room temperature (23 °C). (C) Delivery of drugs using SNAP-PEG-coated dental floss involves a gradual transfer of SNAP from the floss onto the surfaces of teeth and the gingiva within the subgingival region during the flossing process. This results in the deposition of the coating into the periodontal pocket, enabling the sustained release of NO over an extended period.

Dental floss is commonly used to clean between teeth and gingiva, but its effectiveness in consistently reducing plaque and gingiva inflammation, even when used with toothbrushing, is often inconsistent due to its reliance on proper mechanical action.^43,44^ To address this issue, there is a need to enhance the functionality of dental floss by incorporating therapeutic agents that can actively target and eliminate bacteria responsible for plaque formation, going beyond its mechanical function. This can result in more effective plaque control and reduce the risk of dental issues such as cavities and periodontal disease. To address this challenge, an antimicrobial coating containing nitric oxide (NO) donor SNAP and PEG polymer (SNAP-PEG) was devised to facilitate the release of antibacterial NO into the periodontal pocket, presenting an inventive approach to prevent microbial infections (Figure 1A). Polyethylene glycol (PEG) was chosen as the base material for this coating due to its excellent biocompatibility and its ability to dissolve in a variety of solvents. It has been demonstrated in previous research that incorporating SNAP into a PEG matrix results in a composite coating capable of releasing NO under physiological conditions.^45^ In this study, high-molecular-weight PEGs were employed as they solidify at room temperature once the methanol solvent evaporates. This characteristic allows for the development of consistent coatings on a range of substrates (Figure 1B). To illustrate the tunability of NO release rates, different weight percentages of SNAP (1, 5, and 10 wt %) were added to the PEG-methanol mixture. Subsequently, this SNAP-PEG blend was coated on a commercially available nylon dental floss using a simple dip-coating process. This integration of NO-releasing properties into the dental floss transforms it into a viable carrier for delivering NO donors to the subgingival region (Figure 1C). Since topical drug delivery into the periodontal pocket to treat periodontitis is a major challenge due to the limited tissue contact, NO-releasing floss, with its antimicrobial properties, is anticipated to combat periodontitis by eradicating bacteria responsible for biofilm formation without the need for a medical professional. This innovation has the potential to decrease the occurrence of oral health problems and, in turn, lower medical costs. However, future in vivo studies should be implemented to validate the deposition of the floss coating into the periodontal pocket.

### 3.2 Surface Characterization of NO-Releasing Dental Floss

#### 3.2.1 Determination of SNAP Payloads on Surface

**Figure 2:**
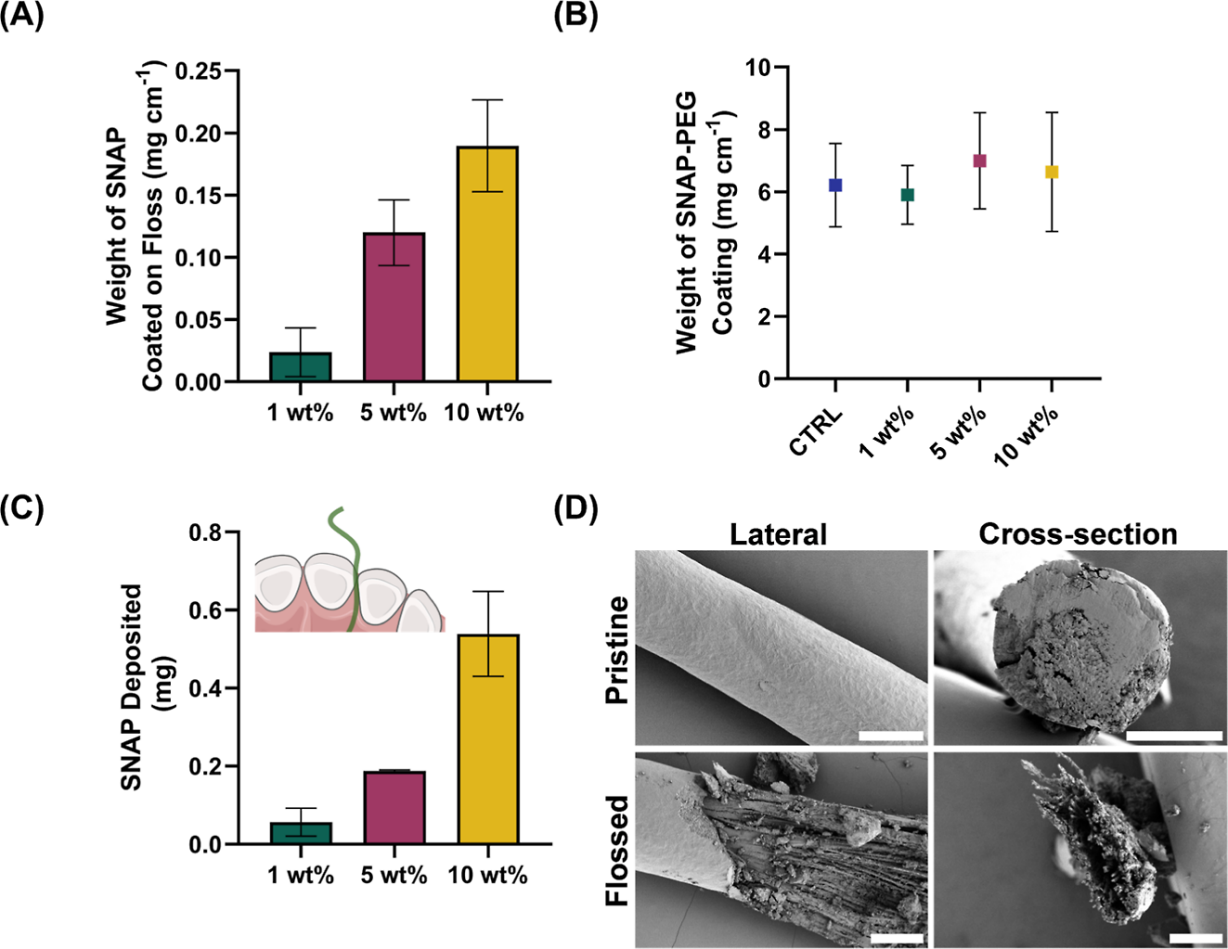
(A) UV–vis spectroscopy absorption data were used to determine the concentration of each weight percent formulation incorporated (*N* = 5). (B) Determination of uniformity of the SNAP-PEG-coated NO-releasing floss to illustrate the uniformity in the fabrication (*N* = 16). An indirect drug efficiency study (C) drug delivery efficiency of SNAP-PEG-coated floss in depositing the coating between the teeth of a tooth model. (D) SEM Images show the morphology of the 1 wt % SNAP-PEG coating on floss before and after deposition of the coating in a tooth model. The scale bars represent 500 μm.

The NO donor SNAP was loaded in the PEG matrix by adding varying payloads (1, 5, and 10 wt %) during the fabrication process. The SNAP-PEG coating was then dip-coated onto the floss’s surface and left to dry at room temperature (RT), facilitating the evaporation of methanol and the formation of a consistent coating. The amount of SNAP embedded into the coating was determined to evaluate the coating’s ability to work as a targeted drug delivery system (Figure 2A). To demonstrate how much SNAP is loaded into each sample formulation per centimeter of floss to understand the distribution of the bioactive material on the floss and ensure SNAP is not aggregating in the PEG coating causing an uneven distribution. The amount of SNAP in the coating was calculated via UV–vis spectroscopy by dissolving 1 cm sections of floss in 1 mL of PBS buffer containing 100 μM EDTA. Results from the study unveiled that the SNAP-PEG coating contained 0.089 ± 0.015, 0.585 ± 0.028, and 0.771 ± 0.081 mg of SNAP per cm of floss for 1, 5, and 10 wt %, respectively. The coating had an increasing weight of SNAP embedded in the PEG matrix with an increasing weight percentage of SNAP added, showing that there was a range of SNAP concentrations stable in the PEG matrix. The coating demonstrated an increasing SNAP content within the PEG matrix as the weight percentage of SNAP added increased. Notably, while a small portion of SNAP underwent minimal degradation during the fabrication steps due to the presence of methanol and the drying process, bioactive levels of SNAP were still present on the floss. This study underscores the tunability of SNAP concentrations within the SNAP-PEG coating, an essential characteristic for achieving targeted drug delivery to the periodontal pocket.

The effectiveness of the SNAP-PEG coating as a precise drug delivery system relies on the uniformity of its application to the nylon floss substrate. To assess this uniformity, measurements were taken of the floss’s weight both before and after the deposition of the SNAP-PEG coating, and the weight of the coated floss was then subtracted from the weight of the nylon core (Figure 2B). The total weight of the coating was measured over three batches of samples among the different formulations to show that the total SNAP-PEG coating was uniform on the floss and did not have a large variation in deposition of the coating on the floss. The average weights of the PEG coatings were found to be 6.211 ± 1.334, 5.899 ± 0.945, 6.992 ± 1.545, and 6.637 ± 1.912 mg cm^−1^ for the control (PEG without SNAP), 1, 5, and 10 wt % coatings, respectively. There were no significant differences observed among the average coating weights. These results highlight the efficiency and consistency of the coating process, particularly in the context of a rapid and straightforward fabrication method. Uniformity in the coated floss is of paramount importance for delivering consistent amounts of SNAP into the periodontal pocket. The study affirms the presence of such uniformity in SNAP-PEG coatings deposited onto the surface of the nylon floss, irrespective of the formulation used.

#### 3.2.2 Indirect Drug Delivery Efficiency of SNAP-PEG-Coated Floss

To assess how easily the SNAP-PEG coating could be deposited in the subgingival region, an indirect drug delivery efficiency test was utilized. The drug delivery efficiency was estimated by flossing a malleable tooth model with SNAP-PEG-coated floss and swabbing the tooth and periodontal pocket to collect the SNAP-PEG coating deposited (Figure 2C). The deposited coating was then dissolved in 1 mL of PBS buffer containing 100 μM EDTA and the weight of SNAP was calculated by reading the absorbance with UV–vis spectroscopy. The results of the indirect drug delivery study indicated that, after flossing, 0.057 ± 0.036, 0.187 ± 0.003, and 0.539 ± 0.109 mg of SNAP were deposited for 1, 5, and 10 wt % samples, respectively, into the periodontal pocket of the tooth model after flossing. A direct correlation was evident between the amount of SNAP deposited in the subgingival region and the SNAP content within the SNAP-PEG coating. Notably, regardless of the weight percentage formulations, the SNAP-PEG-coated dental floss effectively delivered SNAP into the periodontal pocket of the model. These findings provide conclusive evidence of the SNAP-PEG-coated floss’s capability to deliver precise amounts of SNAP into the periodontal pocket.

This approach underscores the precision with which SNAP-PEG-coated dental floss can channel the NO donor specifically into the dental cavity. This offers significant advantages for the targeted administration of antibacterial agents while minimizing systemic drug absorption. By directing NO precisely to the affected area through targeted delivery, the healing process can be significantly enhanced, providing a direct supply of NO to the infection site or damaged region. It is important to note that the quantity of SNAP within the PEG formulation can be customized based on the severity of the condition and the patient’s requirements. Consequently, this methodology illustrates the potential for controlled and localized antimicrobial agent deposition offering versatile applications for the prevention of periodontitis.

#### 3.2.3 Surface Analysis Using Scanning Electron Microscopy and Energy-Dispersive X-ray Spectroscopy

Scanning electron microscopy imaging was used to demonstrate how the coating is effortlessly inserted into the periodontal pocket through direct contact and minimal exertion (Figure 2D). The images show that the coating is relatively uniformly distributed on the floss, corroborating the weight distribution data. After the coated floss samples were lightly mechanically flossed three times in either incisor of the tooth model, the fibers of the floss were then visible. The SEM imaging provided visual evidence that a substantial amount of the coating was deposited into the periodontal pocket, as seen in the flossed structure of the dental floss. It was also observed that the dental floss maintained its original physical attributes, ensuring that it could continue to function effectively as a tool for oral care while incorporating the beneficial properties of the SNAP-PEG coating. An excess amount of coating is applied to the floss to ensure thorough deposition within the oral pocket. Excessive microbial activity can lead to the development of pathological pockets around affected teeth, and this bioactive coating is designed to mitigate subgingival infections commonly associated with periodontal disease. Full EDS spectra are provided in Figure S2 of a 10 wt % SNAP-PEG-coated floss sample. EDS was used for targeted analysis of sample surfaces to find evidence of SNAP and the parent thiol on the surface of the sample. Nitrogen, silicon, sulfur, oxygen, and carbon were evident on the surface of the sample.

### 3.3 NO-Release Kinetics under Physiological Conditions

**Figure 3:**
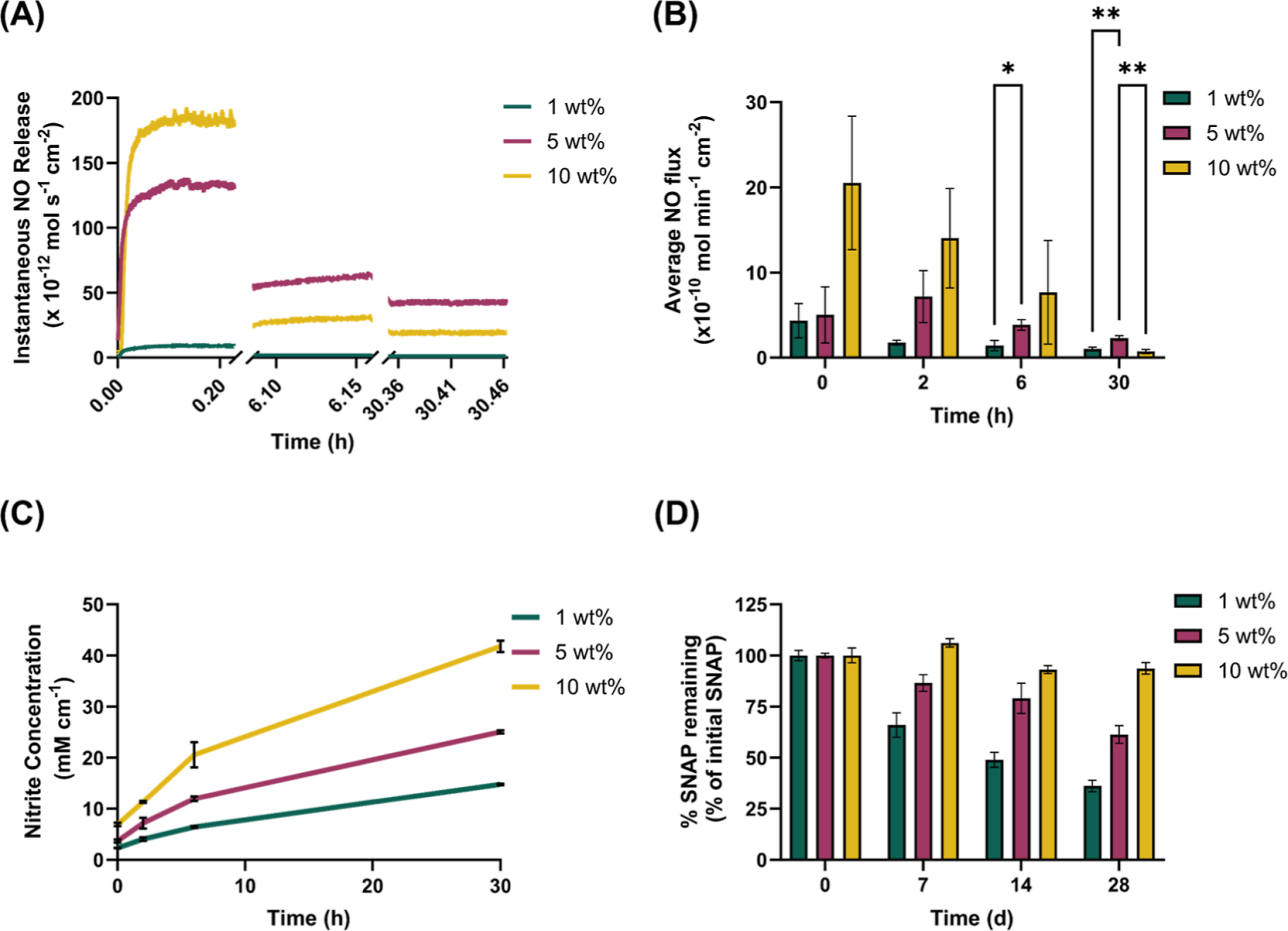
(A) Representative instantaneous NO release profiles of each weight percent of SNAP-PEG coating over a 30 h period (B) average NO flux recorded from 1, 5, and 10 wt % of SNAP-PEG coated dental floss at varying time points (0, 2, 6, and 30 h) reported as the mean ± SD determined using nitric oxide analyzer (*N* = 3). (C) The total nitrite concentration was used to estimate the NO-release in aqueous solution reported as the mean ± SD (*N* = 4). (D) Storage stability analysis of NO-releasing SNAP-PEG-coated floss over 28 days at room temperature (*N* = 3).

NO-releasing hydrogels have previously been made for wound healing and antimicrobial effects with different mechanical properties to have a controlled release of NO.^46^ SNAP is a synthetic tertiary RSNO making it more stable than most endogenous primary RSNOs because of the steric hindrance of the sulfur atom.^47^ SNAP releases NO via thermal decomposition, metal ion catalysis, and photolysis when the light energy corresponds with the SNAP absorption bands at 340 and 590 nm.^23^ To mimic the environment of the oral cavity, the NO release studies were conducted under humidified conditions, ensuring that the coating remained intact at each time point for a comprehensive assessment of the NO release characteristics without immediate dissolution. The magnitude of the NO release from the samples was found to be influenced by the concentration of SNAP embedded in the polymeric matrix. The average flux for each weight percentage (wt %) of SNAP measured depicted the NO release of the floss coating under simulated physiological conditions at various time points up to 30 h (Figure 3A).

Similar to other materials utilizing SNAP as the NO donor, the initial evolution of the NO release exhibited higher levels that gradually decreased over time. This observed rate of NO release can be attributed to the hydrophilic nature of PEG, known for its high-water absorption capacity.^46,48^ Throughout the study, the 10 wt % SNAP samples, on average, demonstrated the highest rates of NO release. The overall trends in the average NO flux were significantly influenced by the weight percentage of SNAP in the PEG coating. By the 30 h time point, the NO release from all samples diminished as the SNAP payload was very minimal (Table S1). Previous research has established a correlation between higher NO flux and increased antibacterial efficiency.^49,50^ Notably, the 10 wt % coatings displayed a higher cumulative average flux compared to the 1 and 5 wt % coatings (Figure 3B). However, there was no significant difference in the average NO flux between the 5 and 10 wt % coatings at 6 h. The profiles representing instantaneous flux at 6 and 30 h for 5 wt % SNAP were greater than those for 10 wt %, which can be explained by the fact that instantaneous flux is representative of a single sample, and there was no significant difference in the average flux at those time points. Furthermore, the NO release demonstrates that the SNAP-PEG coating has the capability to release NO under humid conditions without significant SNAP loss and is able to release bioactive levels of NO, and this release can be sustained for up to 30 h. These data align with previously reported findings with NO-releasing materials containing SNAP, demonstrating NO levels on par with those obtained for the SNAP-PEG coating.^27^

Separate controlled release degradation studies in aqueous solutions with the coated floss samples were completed under static incubation at 37 °C to estimate the NO release with the total nitrite concentration from a Griess assay. The nitrite accumulation test is distinct from a SNAP accumulation study and estimates the NO release rather than the amount of SNAP leached into the solution. The samples were quickly dissolved into PBS with a 100 μM EDTA solution, where it would continue to release NO in solution. It was observed that the total nitrite concentration had a similar release profile to the humid conditions detected for each time point up to 30 h (Figure 3C). This pattern aligns with the NO release patterns observed through the chemiluminescence method, demonstrating the controlled degradation of the coating beyond just humid conditions. These methods described are used to study the NO release profiles in various conditions to which the coating might be exposed after it is deposited on teeth or subgingival tissue.

The SNAP-PEG coating demonstrated controlled release in both solutions in a controlled degradation study and in humid conditions, which can be utilized for targeted drug delivery in the periodontal pocket. Due to SNAP’s degradation under different conditions and NO’s short half-life, effective control of the release conditions from the SNAP-PEG matrix is crucial to harnessing it as a targeted antimicrobial agent within the periodontal pocket. While dental floss is a widely accepted tool for oral hygiene, its potential as a vehicle for topical drug delivery has received limited attention in the literature. Previous research has shown multiple therapeutics can be integrated into the floss for targeted delivery of antibacterial drugs into the periodontal pocket including gold nanoparticles (AuNPs), povidone-iodine, chlorhexidine, etc.^9,51,52^ These approaches have demonstrated effective antibacterial properties, showing promise in combating periodontal infections. However, one key concern with these methods is the stability of the drug coatings over time. Moreover, AuNPs can be expensive to produce, which could increase the overall cost of dental floss products. This may limit their accessibility to a broader population, making it less user-friendly for individuals seeking routine oral care. In contrast, SNAP-PEG-coated dental floss is user-friendly and can easily be integrated into an individual’s daily oral care routine. It is easy to synthesize and economical and offers a unique advantage in terms of stability. The sustained and extended release of NO from the SNAP-PEG coating highlights the long-lasting effectiveness of this material as a therapeutic agent for periodontal care. This characteristic is particularly valuable in the context of periodontal disease management, where continuous and reliable drug delivery is essential for preventing and treating bacterial infections in the oral cavity.

### 3.4 Shelf-Life Stability of SNAP-PEG Coating

The success of biomaterials is highly dependent on their ability to retain function for an extended time after fabrication. To evaluate the duration the SNAP-PEG coating could be stored at room temperature (RT) for effective clinical translation, the percent SNAP remaining was determined with UV–vis spectroscopy after 28 d of storage (Figure 3D). The floss coating was stored in a tightly closed vial, in the dark, and with a desiccant to protect the coating from moisture. Findings from the storage stability analysis indicated that PEG coatings containing higher weight percentages of SNAP exhibited superior retention of the total loaded SNAP compared with coatings with lower weight percentages. For 5 and 10 wt % SNAP samples after the 7 d time point still had greater than 85% of remaining SNAP and 10 wt % had 93.65 ± 2.82% remaining after 28 d. However, the 1 wt % samples degraded more quickly. The 1 wt % coating degraded to 65.94 ± 6.00% remaining over 1 day and went as low as 36.23 ± 2.82 on the 28 d time point. The 10 wt % coating is relatively stable at RT when shielded from light and humid conditions. A limitation for real-world applications is the storage conditions for the floss, as it would require ideal conditions for storage for use including protection from light, heat, humidity, and metal ions for long-term storage.

Previous studies have shown that the incorporation of SNAP into a polymeric matrix enhances its stability as crystalline SNAP embedded in the polymer matrix is released more slowly than dissolved SNAP from the polymer matrix and it shields the RSNO donor from environmental conditions such as light and heat.^26^ NO-releasing materials largely face the challenge of tuning the rate of NO release for their intended purpose as well as maintaining a stable shelf life under relevant medical conditions. However, by incorporating SNAP into the polymeric matrix, the risk of SNAP degradation or loss during storage or application is minimized, allowing for reliable and consistent delivery of the active compound. This increased stability contributes to the overall efficacy and reliability of the floss coating as a drug delivery system for patients.

### 3.5 In Vitro Testing

#### 3.5.1 Antimicrobial Efficacy of SNAP-PEG Coating

**Figure 4:**
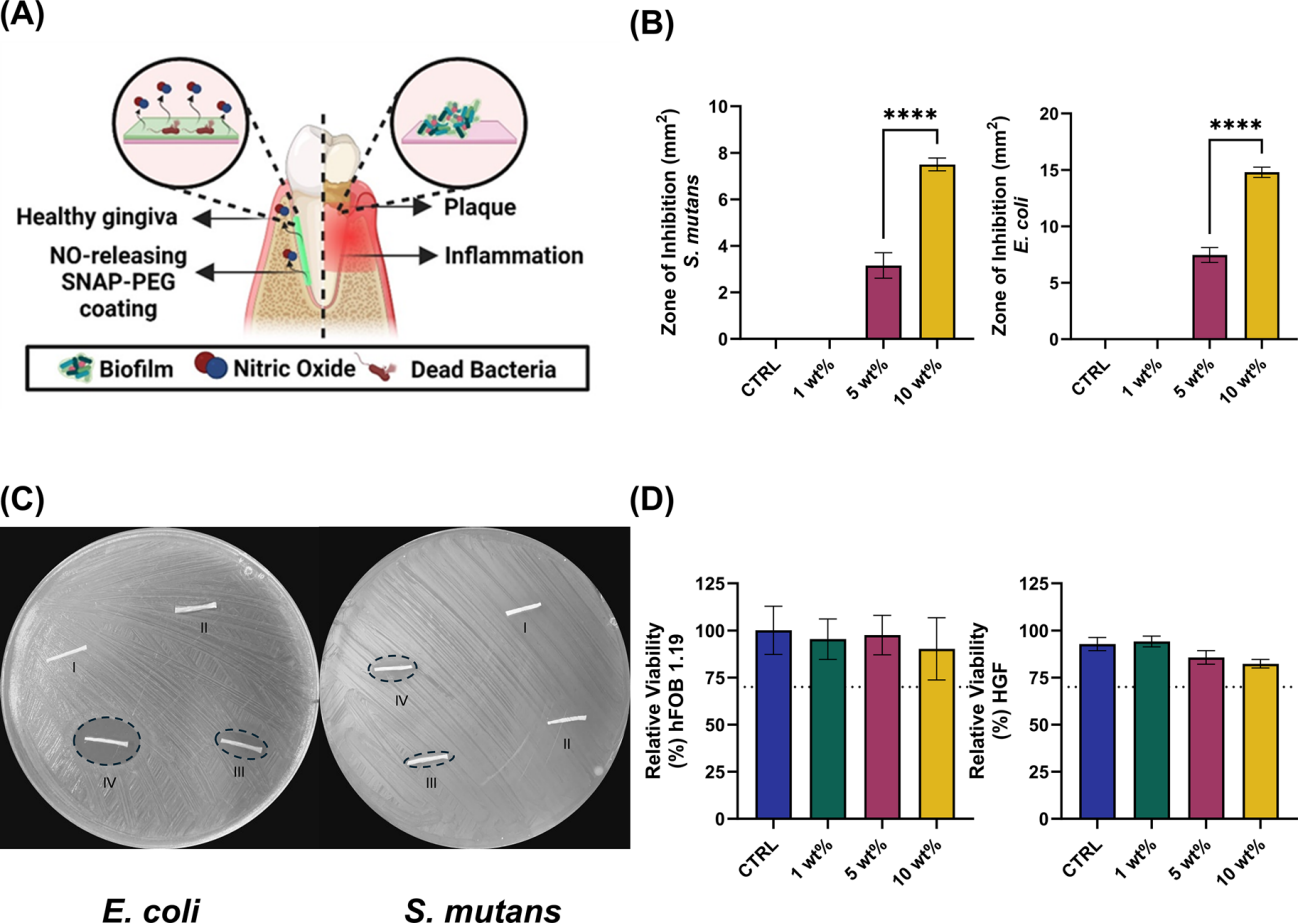
In vitro evaluation of NO-releasing SNAP-PEG coatings. (A) NO-releasing dental floss with inherent broad-spectrum antimicrobial properties can help in the prevention of periodontal infections. (B) Zone of inhibition studies were performed with *S. mutans* and *E. coli*. Final data are shown as mean ± SD (*N* = 5). Statistical significance is presented as ****(*p* <.0001). (C) Representational images of the zone of inhibition on agar plates for *S. mutans* and *E. coli* with the labels I–IV being the PEG control (I), 1 wt % (II), 5 wt % (III), and 10 wt % (IV) (D). Relative cell viability toward hFOB 1.19 and HGF cell lines. Data are presented as the mean percent viability normalized against untreated cells ± SD (*N* = 3).

Periodontitis affects nearly half of the adult population in the United States, and, with a prevalence of about 11.2% worldwide, stands as the sixth most common human disease.^44,53^ This chronic inflammatory condition results in the deterioration of the tissues supporting the teeth and can ultimately lead to tooth loss, affecting speech, nutrition, and aesthetics.^54^ Moreover, periodontitis has broader systemic implications, contributing to conditions like cardiovascular disease, and coronary heart disease, as it fosters systemic inflammation.^55^ The main cause of periodontitis is the buildup of a bacterial plaque biofilm in the subgingival area. This region is the gap between the tooth and the gingiva located beneath the gingival margin. The presence of a bacterial biofilm disrupts the balance in the oral microbiome and triggers a destructive inflammatory immune response from the host. Consequently, plaque removal plays a crucial role in preventing periodonatal associated infections. Furthermore, given the susceptibility of pathogens to re-establish themselves in periodontal pockets, consistent daily plaque removal is essential for an effective treatment regimen. Conventional toothbrushes have limitations in reaching the spaces between teeth, thus requiring the use of specialized tools, such as dental floss, for thorough cleaning of interdental regions. Although dental floss is a common tool in daily oral hygiene routines, it frequently falls short in reducing plaque and mitigating gingival inflammation, even when combined with regular brushing and oral rinsing. This limited efficacy is primarily attributed to the mechanical nature of dental floss.^43^ To overcome these limitations, a NO-releasing antimicrobial coating was integrated with dental floss for the efficient delivery of NO into the subgingival region (Figure 4A).

To assess its antimicrobial efficacy, the SNAP-PEG coating was subjected to zone of inhibition (ZOI) assays targeting both *S. mutans* (Gram-positive) and *E. coli* (Gram-negative). Notably, *S. mutans*, often present in the periodontal pocket, plays a pivotal role by generating acid that erodes enamel and creates a favorable environment for the colonization of other bacteria.^56^ Similarly, *E. coli* is a facultative anaerobic Gram-negative bacteria found in the complex ecosystem of the oral microbiome, with an ability to produce lipopolysaccharides (LPS) to increase the inflammatory action in the periodontal pocket.^57−59^ Furthermore, recent studies have uncovered a potential association between *E. coli* and osteomyelitis in diabetic patients with aggressive bilateral maxillary necrosis.^40^ Consequently, the efficacy of the SNAP-PEG coating was assessed against both *S. mutans* and *E. coli* using a zone of inhibition analysis aiming to showcase its broad-spectrum antibacterial effects.

The results from the ZOI test of the SNAP-PEG coating unveiled a significant difference in the diameter of the inhibited zone of growth for each tested formulation containing 1, 5, and 10 wt % of SNAP, and no zone of inhibition was observed for the control PEG sample, which was expected since the PEG control coating lacked any inherent antibacterial mechanism of action. The lack of a zone and the subsequent absence of antibacterial activity in the control PEG samples confirmed that the observed antibacterial effects were primarily attributed to the presence of SNAP in the coating. The increased zone of inhibition was observed to be directly proportional to the increasing concentration of SNAP present in the coating (Figure 4B,C). On average, the ZOI against *S. mutans* for the 5 wt % samples was 3.2 ± 0.54 mm^2^ and 7.5 ± 0.28 mm^2^ for 10 wt %. Similarly, the ZOI for *E. coli* was 7.5 ± 0.67 mm^2^ for 5 wt % and 14.8 ± 0.46 mm^2^ for 10 wt %. The ZOI for *S. mutans* against the 10 wt % SNAP-PEG coating had approximately 2× more than the 5 wt % (*p* < 0.05). Comparatively, the zone of inhibition for *E. coli* against the 10 wt % SNAP-PEG coating had approximately 2x and greater ZOI than the 1 and 5 wt %, respectively (*p* < 0.05). The antibacterial assay results align with the NO release data from each formulation (Figure 3B), showing a direct correlation between higher SNAP concentrations and increased levels of NO release. Increased NO release results in elevated levels of reactive oxygen species (ROS) within the bacterial environment, triggering processes such as membrane disruption, DNA lysis, and lipid peroxidation, ultimately leading to bacterial death. These data also support previously published research demonstrating the broad-spectrum antimicrobial properties of NO.^60^

NO’s gaseous nature facilitates easy penetration of bacterial membranes, damaging DNA and inactivating heme proteins involved in signal transduction.^61^ Unlike specific antibiotics that target distinct pathways in different bacteria types, NO’s multimechanistic and nonspecific action remains effective across all bacteria types.^30^ Notably, NO exhibits potent antibacterial effects against a wide range of bacteria including both antibiotic-resistant and susceptible bacteria without inducing NO resistance.^62,63^ This surpasses the effectiveness of alternative antibacterial agents, such as gold nanoparticles and chlorhexidine, which are frequently integrated into dental floss for the management of periodontal infections.^9,51,52^ Synthetic chemicals, such as chlorhexidine, may disrupt the natural oral microbiome equilibrium and have cytotoxic effects.^64^ Conversely, NO is a naturally found molecule responsible for several regulatory functions in oral health.

The application of SNAP-PEG-coated dental floss is poised to impede the formation of a biofilm and counteract bacterial proliferation during its early stages, effectively mitigating severe biofilm accumulation within periodontal pockets. This strategy holds particular importance, as biofilm, once established, becomes highly resilient and resistant to conventional treatments, rendering eradication notably challenging. By intervention early with NO-releasing floss, the initial stages of biofilm formation can be disrupted, thereby preventing its establishment and subsequent progression. Consequently, NO-releasing materials offer a multitude of advantages over conventional chemical agents for managing periodontal disease. The controlled release of NO from SNAP-PEG-coated dental floss exemplifies adjustable drug loading and potent antibacterial properties, rendering it a user-friendly approach that could enhance patient access to treatment.

#### 3.5.2 Cytocompatibility of NO-Releasing Floss

Ensuring the SNAP-PEG coating does not induce a cytotoxic response is as important as the level of antimicrobial activity for the goal of preventing periodontal infection propagation. A controlled degradation study for relative cell viability was conducted to determine the compatibility of the different SNAP-PEG coatings under extraction conditions. Two cell types were used, HGFs and hFOB 1.19’s, with the results of contact testing summarized in Figure 4D. HGFs are the most abundant structural cells in the periodontal pocket, being the foundational cells of connecting tissue and playing critical roles in inflammatory processes and wound healing.^65^ Osteoblastic cells are responsible for both soft and hard tissue restoration needed for the treatment of periodontitis. Both cell types showed a relative cell viability greater than 70%, represented by the dashed line, showing a broad cytocompatibility of the material. Similar to other NO-releasing materials for dental applications capable of eradicating dental pathogens the SNAP-PEG floss is effective at concentrations not diminishing the viability of human gingival fibroblast cells.^33^ These results follow previous studies of NO-releasing materials, as it is known that low dosages of NO promote fibroblast proliferation and migration.^66^ Minimizing acute adverse biological effects from leachates from tissue-contacting materials is crucial to prevent further inflammation. This demonstrates the biocompatible properties of the SNAP-PEG floss and, combined with the antimicrobial properties of the floss, illustrates a promising material coating for preventing periodontitis.

## 4 Conclusions

Periodontitis requires continuous maintenance by both patients and health professionals. Despite extensive research, existing approaches fall short of preventing the condition. To address these challenges, a simple and effective floss coating was fabricated. Introducing the advancement to patient care to improve patient compliance as well as the delivery and retention of SNAP into the subgingival region. The SNAP-PEG-coated floss was able to effectively treat both Gram-negative and Gram-positive bacteria commonly present in periodontal tissue. The facile method to fabricate the coating includes the use of a NO donor, SNAP, which is activated by physiological temperature to eliminate bacteria that contribute to periodontal disease. The NO donor SNAP is embedded into a PEG mixture at varying concentrations for a tunable and controlled NO release for 30 h. Among the different concentrations of SNAP incorporated into the PEG coating, the NO flux was determined using a chemiluminescence NO analyzer. The average flux was as high as 23.4 ± 8.34 NO flux (×10^–10^ mol min^–1^ cm^–2^) and had NO release for 30 h illustrating the longevity of the material for flossing. The NO flux levels for the novel SNAP-PEG coatings were antimicrobial-relevant and shown to be a controlled release targeted for the periodontal pocket. Additionally, the antibacterial activity and cytocompatibility were measured in vitro. The SNAP-PEG coating exhibited broad-spectrum antimicrobial action against both *S. mutans* (Gram-positive) and *E. coli* (Gram-negative) in the zone of inhibition studies. All formulations of the SNAP-PEG coating tested were deemed cytocompatible against HGF and hFoB cells, as shown in a controlled release compatibility study. The coating presented increases accessibility to dental care for patients and minimizes the need for mechanical force required to floss for those with lower dexterity with a bioactive additive. The increased accessibility to dental care the coating presents is expected to significantly minimize the heavy financial burden of periodontal diseases. The coating technology can also be applied for different applications, including dental sutures, and other interdental instruments such as interproximal brushes, as the coating is independent of the substrate.^67^ The ease of synthesis and fabrication, capacity of the tunable NO release and coating deposition, antimicrobial activity, cytocompatibility, and shelf-life stability make the SNAP-PEG floss coating a promising new solution for the prevention of periodontal infections and the need for surgical intervention. Additional antimicrobial studies involving anaerobic bacteria are in progress to demonstrate the antibacterial efficacy of the floss within the periodontal pocket against more complex biofilms.

## Supporting Information Available

^1^H NMR spectra of *N*-acetylpenicillamine (NAP) and *S*-nitroso-*N*-acetylpenicillamine (SNAP). Energy dispersive X-ray spectroscopy (EDS). Table of average NO flux (×10^–10^ mol min^–1^ cm^–2^) (PDF)
