# Supplementary material for: Engineering Nitric Oxide-Releasing Antimicrobial Dental Coating for Targeted Gingival Therapy
Source: ACS Appl Bio Mater. 2024 Apr 9;7(5):2993–3004. doi: 10.1021/acsabm.4c00051 (PMC11110066; doi:10.1021/acsabm.4c00051)
Supplement: Supplementary file 1 — mt4c00051_si_001.pdf [file mt4c00051_si_001.pdf]

Supporting Information

Engineering Nitric Oxide-releasing Antimicrobial Dental Coating for Targeted Gingival Therapy

Manjyot Kaur Chug, [1†] Natalie Crutchfield, [1†] Mark Garren,[1] Hitesh Handa,[1,2] Elizabeth J. Brisbois[1]*

† Authors declare equal contribution to this work.

[1] School of Chemical, Materials & Biomedical Engineering, College of Engineering, University of Georgia, Athens, Georgia, USA

[2] Department of Pharmaceutical and Biomedical Sciences, College of Pharmacy, University of Georgia, Athens, Georgia, USA

* Correspondence

Elizabeth J. Brisbois, Ph.D.

School of Chemical, Materials, and Biomedical Engineering

University of Georgia

302 E Campus Rd

Athens, GA 30605

Phone: 706-542-1243

E-mail: ejbrisbois@uga.edu

**Contents**	**Page**
**Figure S1.** ^{1}H NMR spectra of N-acetylpenicillamine (NAP) and S-nitroso-N-acetylpenicillamine (SNAP)	S-3
**Figure S2.** Energy dispersive X-ray spectroscopy (EDS)	S-4
**Table S1.** Table of Average NO flux (x 10^{-10} mol min^{-1} cm^{-2})	S-5

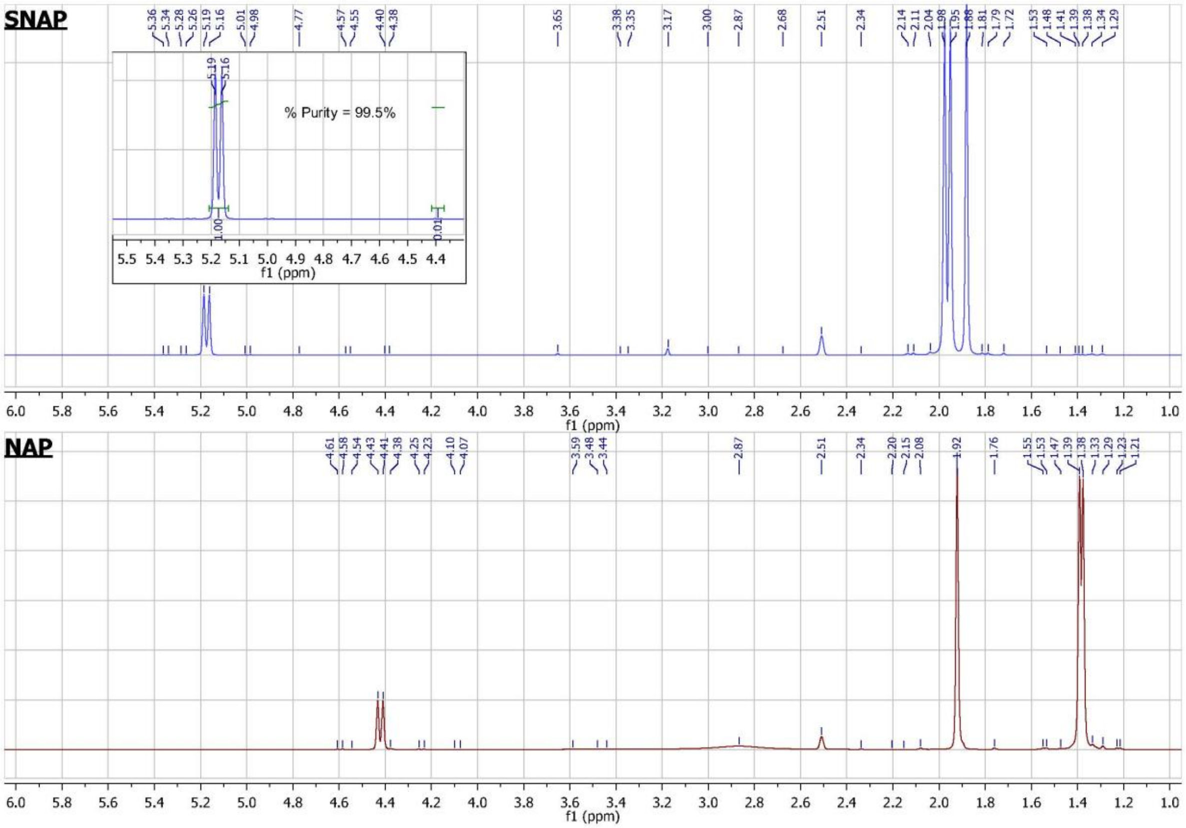

Figure S1. ^{1}H NMR spectra of N-acetylpenicillamine (NAP) and S-nitroso-N-acetylpenicillamine (SNAP) to confirm structure and purity of the RSNO donor.

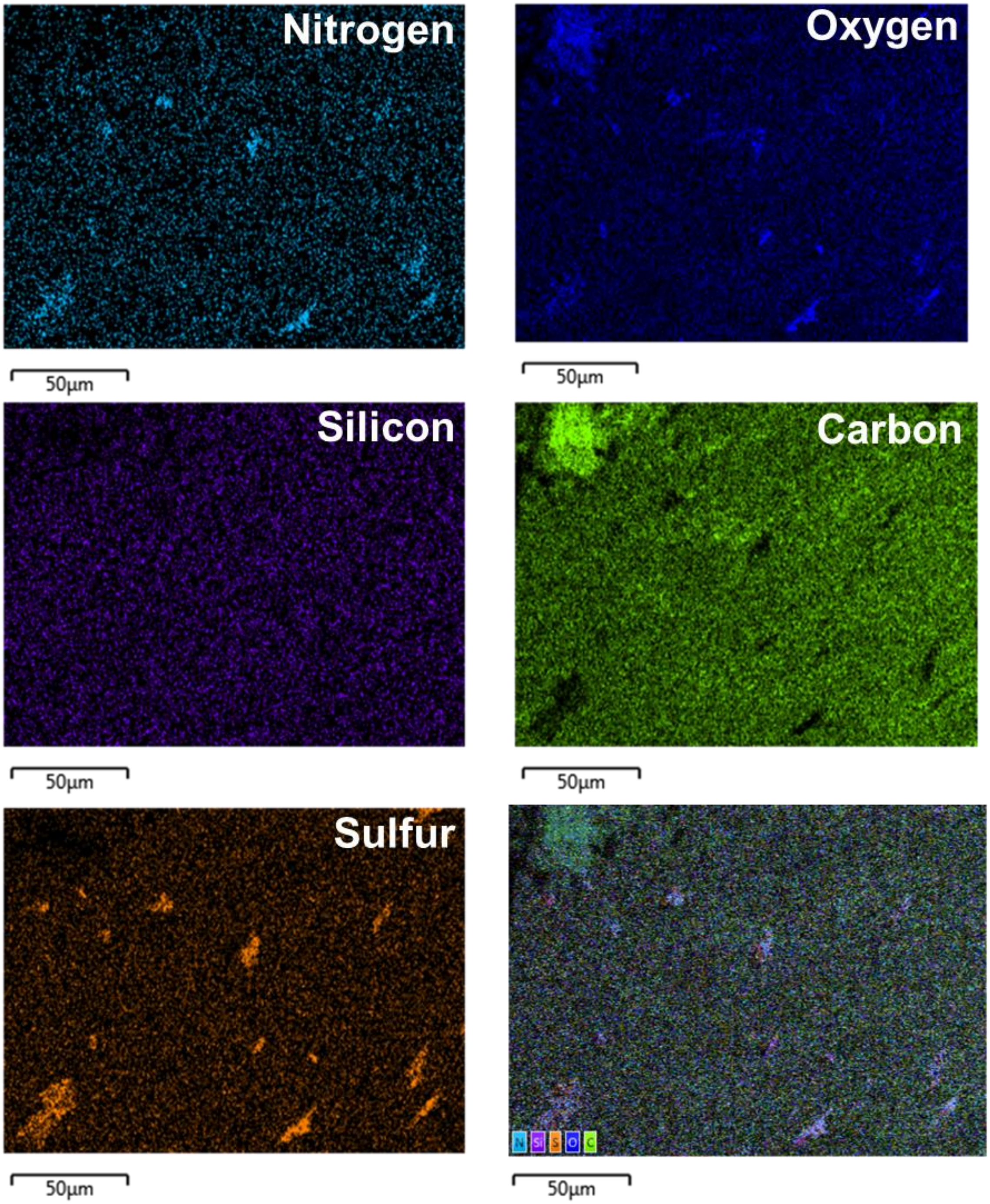

Figure S2. Energy dispersive X-ray spectroscopy (EDS) was used for a targeted analysis of sample surfaces to find evidence of *S*-nitroso-*N*-acetylpenicillamine (SNAP) on the surface of coating. Nitrogen, silicon, sulfur, oxygen, and carbon were evident on the surface of the sample. The images are on the 10 wt.% SNAP-PEG coated floss.

SNAP wt. % in PEG	Average NO flux (x 10^{-10} mol min^{-1} cm^{-2}) Time (h)			
	**0**	**2**	**6**	**30**
**1 wt%**	3.97 ± 1.79	1.45 ± 0.19	1.19 ± 0.37	0.91 ± 0.20
**5 wt%**	4.88 ± 2.70	6.89 ± 2.49	3.61 ± 0.43	2.21 ± 0.22
**10 wt%**	23.4 ± 8.34	14.0 ± 4.78	7.68 ± 4.95	0.74 ± 0.19

Table S1 – Average NO flux data over 30 h at different time points for each weight percentage of SNAP in the floss coatings.
